# Antimicrobial resistance patterns of Pseudomonas aeruginosa isolated from canine clinical cases at a veterinary academic hospital in South Africa

**DOI:** 10.4102/jsava.v91i0.2052

**Published:** 2020-09-22

**Authors:** Ulemu L. Eliasi, Dikeledi Sebola, James W. Oguttu, Daniel N. Qekwana

**Affiliations:** 1 Section Veterinary Public Health, Department of Paraclinical Science, Faculty of Veterinary Sciences, University of Pretoria, Pretoria, South Africa; 2 Department of Agriculture and Animal Health, College of Agriculture and Environmental Sciences, University of South Africa, Johannesburg, South Africa

**Keywords:** antimicrobial resistance, *Pseudomonas aeruginosa*, dogs, multi-drug resistance, veterinary

## Abstract

Although *Pseudomonas aeruginosa (P. aeruginosa)* can infect both animals and humans, there is a paucity of veterinary studies on antimicrobial resistance of *P. aeruginosa* in South Africa. Secondary data of canine clinical cases presented at the hospital from January 2007 to December 2013 was used. The following information was recorded: type of sample, the date of sampling and the antimicrobial susceptibility results. Frequencies, proportions and their 95% confidence intervals were calculated for all the categorical variables. In total, 155 *P. aeruginosa* isolates were identified and included in this study. All the isolates were resistant to at least one antimicrobial (AMR), while 92% were multi-drug resistant (MDR). Most isolates were resistant to lincomycin (98%), penicillin-G (96%), orbifloxacin (90%), trimethoprim-sulfamethoxazole (90%) and doxycycline (87%). A low proportion of isolates was resistant to imipenem (6%), tobramycin (12%), amikacin (16%) and gentamicin (18%). A high proportion of MDR-*P. aeruginosa* isolates was resistant to amoxycillin-clavulanic acid (99%), tylosin (99%), chloramphenicol (97%) and doxycycline (96%). Few (6%) of MDR-*P. aeruginosa* isolates were resistant to imipenem. *Pseudomonas aeruginosa* was associated with infections of various organ systems in this study. All *P. aeruginosa* isolates of *P. aeruginosa* exhibited resistance to β-lactams, fluoroquinolones and lincosamides. Clinicians at the hospital in question should consider these findings when treating infections associated with *P. aeruginosa*.

## Background

*Pseudomonas aeruginosa* (*P. aeruginosa*) is a gram-negative, saprophytic and opportunistic pathogen capable of infecting both humans and animals (Alhazmi 2015). The organism is ubiquitous in moist environments such as water and soil (Iregbu & Eze 2015).

In human medicine, *P. aeruginosa* has been associated with nosocomial infections of the urinary tract, surgical wounds and bloodstream (Peleg & Hooper 2010). In addition, the organism has been isolated in patients with severe burn wounds, meningitis, brain abscesses and other underlying clinical conditions (Hauser & Ozer 2011; Strateva & Yordanov 2009; Türkyilmaz 2008). In veterinary medicine, *P. aeruginosa* has been isolated from dogs with chronic otitis externa, pyoderma, conjunctivitis, septicaemia, lower urinary tract infections, pneumonia and bacterial endocarditis (Dégi, Cristina & Stancu 2010; Petrov et al. 2013). Dogs with compromised immune systems and co-morbid conditions are at a higher risk of *P. aeruginosa* colonisation (Musser & Beamer 1961).

Although *P. aeruginosa* infections in human medicine are well documented in South Africa (Mudau et al. 2013; Odjadjare et al. 2012; Perovic et al. 2008), studies of *P. aeruginosa* infections in veterinary medicine could not be sourced. This is despite *P. aeruginosa* organisms having been reported as having high levels of resistance to commonly used antimicrobial agents such as penicillins, tetracyclines, fluoroquinolones and aminoglycosides (Prescott et al. 2003; Vingopoulou et al. 2018).

This study investigated the antimicrobial resistance patterns of *P. aeruginosa* from clinical samples obtained from dogs presented to a veterinary academic hospital in South Africa between January 2007 and December 2013. The results of this study will help guide empirical antimicrobial selection for the treatment of dogs infected with *P. aeruginosa* in veterinary medicine. In addition, the information generated from this study will contribute to antimicrobial resistance surveillance programmes in animal health.

## Methods

### Study area

This study was conducted at a veterinary academic hospital located in Pretoria, South Africa. The veterinary hospital provides services for multiple veterinary disciplines, including internal medicine and surgical procedures. It offers training in companion, livestock and wildlife studies, and serves as a referral centre for complicated medical and surgical cases from other parts of the city, country and neighbouring countries.

### Data collection

This study used secondary data of *P. aeruginosa* clinical isolates from dogs admitted to the veterinary academic hospital between January 2007 and December 2013. The hospital requires clients to sign consent forms granting the hospital permission to use information obtained from their animals for purpose of teaching and research. Information such as patient unique number, type of sample, date of sample collection, bacterial culture and antimicrobial susceptibility of the isolates was extracted from paper records submitted during the study period. The records of all the patients that yielded samples positive for *P. aeruginosa* (*n* = 155) were reviewed and included in this study.

### Bacterial isolates and antimicrobial susceptibility testing

The bacteriology laboratory cultures all the submitted clinical samples to isolate *P. aeruginosa* using standard bacteriological methods as described by Quinn et al. (1994). Isolates were then subjected to a panel of 19 antimicrobial agents using the disk diffusion method to establish their susceptibility profiles. The bacteriology laboratory follows the Clinical Laboratory Standards Institute guidelines (Clinical Laboratory Standards Institute 2007, 2008, 2009, 2010, 2011, 2012) to isolate and conduct antimicrobial susceptibility testing.

Antimicrobials included in the test panel were the following: 30 *µ*g-amikacin, 20/10 *µ*g ampicillin, 100 *µ*g carbenicillin, 30 *µ*g ceftazidime, 30 *µ*g cephalothin, 30 *µ*g chloramphenicol, 30 *µ*g doxycycline, 5 *µ*g enrofloxacin, 10 *µ*g gentamicin, 30 *µ*g imipenem, 30 *µ*g kanamycin, 2 *µ*g lincomycin, 100 *µ*g Lincospectin, 5 *µ*g orbifloxacin, 10 *µ*g penicillin-G, 100 *µ*g piperacillin, 25 *µ*g trimethoprim-sulfamethoxazole, 20/10 *µ*g amoxycillin-clavulanic acid, 10 *µ*g tobramycin and 15 *µ*g tylosin.

The laboratory classifies the results of the antibiogram as intermediate, sensitive or resistant, following the Clinical and Laboratory Standards Institute guidelines (Clinical and Laboratory Standards Institute 2007, 2008, 2009, 2010, 2011, 2012). However, for this study, isolates that had been classified as having intermediate susceptibility were reclassified as being resistant. Multi-drug resistance (MDR) was defined as resistance to at least one antimicrobial in three or more antimicrobial categories (Magiorakos et al. 2011).

Antimicrobial agents such as penicillins, cephalosporins, aminoglycosides and sulfamethoxazole-trimethoprim, which the organism showed inherent resistance to, were excluded from the MDR analysis (Pang et al. 2019). Lincospectin and lincomycin were also removed from the analysis because they are mainly efficacious against gram-positive bacteria (Farrington 2012). However, the newer generation β-lactams (imipenem) were included in the analysis because they have a broad-spectrum activity that allows them to be active against gram-negative organisms. Furthermore, amoxycillin-clavulanic acid was included in the calculation of MDR because clavulanic acid was shown to be effective against beta-lactamase enzymes.

### Data management and analysis

The dataset was assessed for duplicates and missing information such as the lack of antibiogram results. None of the isolates had missing information and there were no duplications in the dataset.

All statistical analyses were performed using Statistical Package for Social Sciences (SPSS) (IBM SPSS statistics version 25). ‘Specimen types’ with a frequency of less than 4% were recategorised into a new category called ‘others’. Thus, the category ‘others’ included specimen types such as aspirates, semen, prostate fluid, vaginal swabs, lung or pleural fluid, bile samples, foreign-object swabs, bone, nasal swabs, trans-tracheal aspirates and oral-cavity swabs. Descriptive statistics (i.e. frequencies and proportions) were computed and presented using tables.

### Ethical consideration

This article followed all ethical standards for a research without direct contact with human or animal subjects.

## Results

Thirty-four percent (34%, 52/155) of the *P. aeruginosa* isolates included in this study were recovered from ear canal samples followed by urine (22%, 34/155) and skin (10%, 16/155) samples. Abscesses contributed to the lowest proportion of isolates (4%, 6/155). Meanwhile, 30% (46/155) of the positive samples were categorised as ‘others’.

Almost all *P. aeruginosa* isolates included in this study were resistant to lincomycin (98%, 150/153), penicillin-G (96%, 148/154), amoxycillin-clavulanic acid (93%, 142/152), carbenicillin (92%, 93/101), cephalothin (90%, 140/154) and doxycycline (87%, 134/154). However, lower levels of resistance were observed to imipenem (6%, 6/100), tobramycin (12%, 12/96) and gentamicin (18%, 29/154) (Table 1).

**TABLE 1:** Antimicrobial resistance profile of *Pseudomonas aeruginosa* isolates from dog clinical samples tested at a veterinary academic hospital, South Africa.

Antimicrobial category	%	*n*/N	95% CI	
			Lower	Upper
**Aminoglycosides**				
Amikacin	16	26/156	11.64	23.3
Gentamicin	18	29/154	13.44	25.74
Kanamycin	89	134/150	83.38	93.33
Tobramycin	12	12/96	7.29	20.59
**Penicillins**				
Carbenicillin	92	93/101	85. 14	95.93
Penicillin-G	96	148/154	91.76	98.20
Piperacillin	86	80/93	77.54	91.65
Amoxicillin/Ampicillin	92	133/144	86.84	95.68
**Carbapenem**				
Imipenem	6	6/100	2.78	12.48
**Cephalosporins**				
Cephalothin	90	140/154	85.32	94.51
Ceftazidime	77	78/101	68.93	85.00
**Combination**				
Amoxycillin-clavulanic acid	93	142/152	88.31	96.39
**Tetracycline**				
Doxycycline	87	134/154	80.79	91.43
**Amphenicols**				
Chloramphenicol	89	132/148	83.16	93.24
**Fluoroquinolones**				
Orbifloxacin	90	137/152	84.36	93.93
Enrofloxacin	73	113 /154	65.89	79.73
**Macrolide**				
Tylosin-tartrate	92	143/154	87.66	95.96
**Lincosamides**				
Lincomycin	98	150/153	94.39	99.33
**Lincosamide-aminoglycoside**				
Lincomycin- spectinomycin	90	138/153	84.46	93.97

CI, confidence interval.

### Multi-drug resistance

Almost all (92%, 142/155) *P. aeruginosa* isolates were MDR, with a high proportion of these MDR isolates exhibiting resistance to amoxycillin-clavulanic acid combination (99%, 132/133), tylosin (99%, 137/139), chloramphenicol (97%, 130/133) and doxycycline (96%, 134/139). Only 6.06% (6/99) of MDR *P. aeruginosa* were resistant to imipenem (Table 2).

**TABLE 2:** Proportions of various antimicrobials that were involved in the multi-drug resistance combinations.

Antimicrobial agent	%	*n*/N	95% CI	
			Lower	Upper
Chloramphenicol	97.7	130/133	95.2	100
Doxycycline	96.4	134/139	93.3	99.5
Enrofloxacin	78.8	111/139	73.1	86.5
Imipenem	6.0	6/99	1.3	10.7
Orbifloxacin	96.3	132/137	93.2	99.4
Amoxycillin-clavulanic acid	99.2	132/133	97.7	100.0
Tylosin	98.5	137/139	96.5	100.0

CI, confidence interval.

## Discussion

We investigated the antimicrobial resistance patterns of *P. aeruginosa* in samples from canine clinical cases presented at a veterinary academic hospital in South Africa. Most *P. aeruginosa* isolates included in this study were isolated from ear-canal swabs and urine samples. Several other studies have also reported *P. aeruginosa* involvement in otitis externa (Dégi et al. 2010; Mekić, Matanović & Šeol 2011; Pye 2018; Steen & Paterson 2012) and urinary tract infections in dogs (Thompson et al. 2011; Wong, Epstein & Westropp 2015). However, given that *P. aeruginosa* is a secondary pathogen, more clinical and laboratory information is needed to determine the significance of these results (Weese et al. 2019). Although the presence of *P. aeruginosa* at these body sites could be attributed mainly to the increased sensitivity to infection because of the easy access (Cabassi et al. 2017), early diagnosis and implementation of the correct treatment are still important for improved prognosis (Marza et al. 2006).

### Resistance of *Pseudomonas aeruginosa* to β-lactams

We observed that a high proportion (86%) of *P. aeruginosa* isolates was resistant to piperacillin. This is contrary to the 14% piperacillin resistance amongst *P. aeruginosa* isolated from human cancer, burn wounds and cardiac-neuro-paediatric surgical patients observed in Kuwait (Mokaddas & Sanyal 1999). A high proportion (92%) of *P. aeruginosa* resistant to amoxicillin-clavulanic acid was also observed in this study, which is consistent with the observation by Gad, El-Domany and Ashour (2008) who reported 95% amoxicillin-clavulanic acid resistance amongst *P. aeruginosa* isolated from human patients with respiratory tract, urinary tract and skin infections in Egypt. Human studies have also reported high proportions of *P. aeruginosa* isolates resistant to ceftazidime (Khan, Khan & Kazmi 2014; Oliveira et al. 2005; Papp-Wallace et al. 2011; Pintarić et al. 2017). This is consistent with the 77% resistance observed in this study.

The results of this study and other studies suggest that resistance against β-lactams is common amongst *P. aeruginosa* (Ansari et al. 2016; Mishra et al. 2012; Rafiee et al. 2014). This resistance is attributed to intrinsic resistance mediated by low membrane permeability and production of AmpC beta-lactamase amongst *P. aeruginosa* isolates (Pechère & Köhler 1999).

In contrast, we observed low levels (6%) of imipenem resistance amongst *P. aeruginosa* isolates. Our findings are consistent with the 10% imipenem resistance amongst *P. aeruginosa* from dogs with otitis externa in Brazil (Oliveira et al. 2005). In the light of these findings, imipenem remains the most effective drug for the treatment of β-lactam resistant *P. aeruginosa* and would most likely lead to a successful treatment outcome if used to treat *P. aeruginosa* exhibiting MDR at the veterinary hospital under study (Papp-Wallace et al. 2011).

### Resistance to fluoroquinolones and tetracyclines

In this study, a higher proportion of resistance to enrofloxacin (73%) and orbifloxacin (90%) was observed amongst *P. aeruginosa* isolates from dogs as compared to 53% enrofloxacin resistant *P. aeruginosa* isolates from dogs reported by Pintarić et al. (2017). Similarly, Rubin et al. (2008) reported lower proportions of *P. aeruginosa* from canine clinical isolates that were resistant to enrofloxacin (31%) and orbifloxacin (52%). Although we are not able to explain the difference between our results and those of other researchers, the resistance to fluoroquinolone observed in *P. aeruginosa* is generally attributed to the low permeability of the bacteria’s outer membrane that limits the rate of penetration of antibiotic molecules into the cells (Nicas & Hancock 1983).

We also observed a high proportion (87%) of doxycycline resistant *P. aeruginosa*. This is comparable with the 91.07% and 99.6% resistance reported by Javiya et al. (2008) and Shah, Wasim and Abdullah (2015), respectively. Similarly, a high proportion (100%) of doxycycline resistant *P. aeruginosa* isolates from dogs with otitis externa was reported by Petrov et al. (2013). The high level of resistance to doxycycline observed in this study suggests that clinicians at the veterinary academic hospital under study might have to reconsider prescribing doxycycline for the treatment of *P. aeruginosa* infections in dogs presented at this the hospital.

### Resistance to aminoglycosides

In comparison with resistance levels to other drugs observed and discussed above, low resistance levels to amikacin (16%), gentamicin (18%) and tobramycin (12%) were observed amongst *P. aeruginosa* isolates. This is consistent with the findings by Yukawa et al. (2017) who also reported low levels of *P. aeruginosa* resistance to amikacin (2.5%) and gentamicin (4.5%) in clinical cases of dogs and cats in Japan. Khan and Faiz (2016) also reported low proportions of *P. aeruginosa* isolates resistant to amikacin (7.4%) and gentamicin (11.6%) in various human clinical cases in Saudi Arabia. This is contrary to the view of some authors that *P. aeruginosa* tends to exhibit intrinsic resistance to aminoglycosides (Pang et al. 2019). The latter view is supported by studies that have reported very high proportions of *P. aeruginosa* isolates that are resistant to aminoglycosides. For example, Penna et al. (2011) in Brazil reported a high proportion of *P. aeruginosa* isolates from dog clinical cases that were resistant to amikacin (70%), gentamicin(71%) and tobramycin (65%). Javiya et al. (2008) in India also reported high proportions of *P. aeruginosa* isolates from human clinical cases that were resistant to amikacin (50%), gentamicin (67%) and tobramycin (66%). Similarly, 89% of *P. aeruginosa* isolates in this study were resistant to kanamycin. This is comparable with the 90% resistance to kanamycin amongst canine clinical isolates reported by Rubin et al. (2008) in the United States.

The higher proportion of resistance to kanamycin compared with other aminoglycosides that was observed in this study could be attributed to chromosomal aphA-encoded aminoglycoside phosphoryl transferase (APH(3’) IIb), which are enzymes that inactivate the action of antimicrobials, leading to resistance (Morita, Tomida & Kawamura 2013). Therefore, the results of this study support the theory of variations in the susceptibility of *P. aeruginosa* to different aminoglycosides based on their mechanism of action. In view of this, the observations cast doubt on the efficacy of kanamycin in the treatment of *P. aeruginosa* infections amongst clinical cases presented at the veterinary academic hospital (Poole 2005).

Despite the widely accepted view that *P. aeruginosa* exhibits intrinsic resistance to aminoglycosides, available evidence suggests aminoglycosides such as amikacin or gentamicin are useful in the treatment of respiratory infections (Poole 2005). Furthermore, commercial topical preparations for treatment of ear infections also contain aminoglycosides that are known to be effective (Boyd, Santoro & Gram 2019).

### Multi-drug resistance

Overall, 92% of *P. aeruginosa* isolates included in this study were MDR. Several other authors have also reported MDR levels of up to 97.9% amongst *P. aeruginosa* clinical isolates from humans (Shokri et al. 2016). In contrast, other authors have reported low proportions of MDR-*P. aeruginosa,* ranging from 14% to 29% in human studies conducted in Pakistan and Saudi Arabia (Gill et al. 2011; M.A. Khan & Faiz 2016; Tam et al. 2010; Ullah, Malik & Ahmed 2009).

*Pseudomonas aeruginosa* is known to exhibit intrinsic resistance against β-lactams, fluoroquinolones, tetracyclines, aminoglycosides and lincosamides (Iregbu & Eze 2015; Mekić et al. 2011; Steen & Paterson 2012; Türkyilmaz 2008). Intrinsic resistance is caused by factors such as low outer membrane permeability, the production of AmPc β-lactamase and the presence of efflux systems MexA-MexB-OprM, MexC-MexD-OprJ, MexE-MexF-OprN and MexX-MexY-Op (Morita et al. 2001, 2013). Because drugs against which *P. aeruginosa* exhibiting intrinsic resistance were not included in the determination of MDR, the high proportion of MDR-*P. aeruginosa* (92%, 142/155) observed in the present study is most likely explained by acquired resistance. This view is supported by the observation that antimicrobials that were frequently involved in MRD combinations such as enrofloxacin, imipenem, orbifloxacin and amoxycillin-clavulanic acid are drugs that *P. aeruginosa* is known not to exhibit intrinsic resistance to. Furthermore, because the veterinary hospital where the study was conducted is a teaching and referral hospital, it is also possible that by the time most of the dogs from which the samples were collected were presented at the hospital, they would already have been exposed to antimicrobial treatment. It is known that exposure to antimicrobials is a risk factor for the development of resistance. However, it is not possible to confirm this assertion because of lack of information on previous antimicrobial exposure amongst the dogs that were sampled.

### Limitations of the study

The study was limited to only one veterinary academic hospital and did not include isolates from other veterinary hospitals in the vicinity of the study area. In view of this, findings reported in this study cannot be generalised to the whole of the Gauteng province. A history of previous antimicrobial usage amongst the dogs tested was also not available to the researchers; therefore, it was not possible to associate the resistance patterns observed with antimicrobial usage patterns. Because intermediate resistant isolates were reclassified as resistant, there is a possibility that the proportions of resistance observed in this study were slightly overestimated. In addition, the susceptibility testing method used in this study was the Kirby Bauer Disk Diffusion (KBDD) method, which is reported to be limited when compared with the minimum inhibitory concentration (MIC) clinical application. Moreover, topical preparations appear to still be effective against *P. aeruginosa,* despite the KBDD method indicating a higher prevalence of resistance (Boyd et al. 2019). This notwithstanding, the KBDD method still provides accurate susceptibility testing to guide therapeutic options. Therefore, the results of this study contribute to baseline data for establishing the burden and patterns of antimicrobial resistance of *P. aeruginosa* from canine clinical isolates.

## Conclusion

In this study, *P. aeruginosa* was isolated from dogs presented at the veterinary academic hospital with otitis externa, urinary tract infections and some skin infections. Furthermore, a high proportion of *P. aeruginosa* from these clinical cases was MDR. It is evident that *P. aeruginosa* from the study population tends to exhibit high resistance mainly to antimicrobials like β-Lactams, tetracycline, amphenicol, fluoroquinolone, macrolide and lincosamide. However, resistance against imipenem, amikacin, gentamicin and tobramycin in the same population, tends to be lower. In view of this, we recommend that clinicians at the hospital in question should take these findings into consideration when deciding on the treatment for cases associated with *P. aeruginosa* infections. The high level of resistance observed against kanamycin compared with other aminoglycosides in this study supports the theory of differences in *P. aeruginosa* susceptibility patterns within the aminoglycoside antimicrobial category.

## References

[CIT0001] Alhazmi A., 2015, ‘Pseudomonas aeruginosa: Pathogenesis and pathogenic mechanisms’, *International Journal of Biology* 7(2), 44–67. 10.5539/ijb.v7n2p44

[CIT0002] Ansari S., Dhital R., Shrestha S., Thapa S., Puri R., Chaudhary N. et al., 2016, ‘Growing menace of antibacterial resistance in clinical isolates of Pseudomonas aeruginosa in Nepal: An insight of beta-lactamase production’, *BioMed Research International* 2016, 8, Article ID 6437208. 10.1155/2016/6437208 PMC5011509 27642599

[CIT0003] Boyd M., Santoro D. & Gram D., 2019, ‘In vitro antimicrobial activity of topical otological antimicrobials and Tris-EDTA against resistant Staphylococcus pseudintermedius and Pseudomonas aeruginosa isolates from dogs’, *Veterinary Dermatology* 30(2), 139–e40. 10.1111/vde.12717 30672043

[CIT0004] Cabassi C.S., Sala A., Santospirito D., Alborali G.L., Carretto E., Ghibaudo G. et al., 2017, ‘Activity of AMP2041 against human and animal multidrug resistant Pseudomonas aeruginosa clinical isolates’, *Annals of Clinical Microbiology and Antimicrobials* 16(1), 17 10.1186/s12941-017-0193-1 28335779 PMC5364734

[CIT0005] Clinical and Laboratory Standards Institute, 2007, *Performance standards for antimicrobial susceptibility testing: Seventeenth informational supplement*, CLSI document M100-S17, Clinical and Laboratory Standards Institute Wayne, PA.

[CIT0006] Clinical and Laboratory Standards Institute, 2008, *Performance standards for antimicrobial disk and dilution susceptibility tests for bacteria isolated from animals: Approved standard*, 3rd edn., CLSI document M31-A3, Clinical and Laboratory Standards Institute, Wayne, PA.

[CIT0007] Clinical and Laboratory Standards Institute, 2009, *Performance standards for antimicrobial susceptebility testing: Nineteenth informational supplement M100-S19*, Clinical and Laboratory Standards Insitute Wayne, PA.

[CIT0008] Clinical and Laboratory Standards Institute, 2010, *Performance standards for antimicrobial susceptibility testing: Twentieth informational supplement M100-S20*, Clinical and Laboratory Standards Institute Wayne, PA.

[CIT0009] Clinical and Laboratory Standards Institute, 2011, *Performance standards for antimicrobial susceptibility testing: Twenty-first informational supplement: Approved standard*, Clinical and Laboratory Standards Institute Wayne, PA.

[CIT0010] Clinical and Laboratory Standards Institute, 2012, *Performance standards for antimicrobial susceptibility testing: Twenty-second informational supplement. This document provides updated tables for the Clinical and Laboratory Standards Institute antimicrobial susceptibility testing standards M02-A11 and M07*, vol. 32 Clinical and Laboratory Standards Institute, Wayne, PA.

[CIT0011] Degi J., Cristina R.T. & Stancu A., 2010 ‘Otitis externa caused by bacteria of the genus *Pseudomonas* in dogs’, *J Lucrari Stiintifice Medicina Veterinara*, 63(1), 410–415.

[CIT0012] Farrington M., 2012, ‘Antibacterial drugs’, in *Clinical pharmacology*, Morris Brown and Peter Bennett, Churchill Livingstone, 11th edn., pp.173–190, London, United Kingdom.

[CIT0013] Gad G.F., El-Domany R.A. & Ashour H.M., 2008, ‘Antimicrobial susceptibility profile of Pseudomonas aeruginosa isolates in Egypt’, *Journal of Urology* 180(1), 176–181. 10.1016/j.juro.2008.03.081 18499192

[CIT0014] Gill M., Usman J., Kaleem F., Hassan A., Khalid A., Anjum R. et al., 2011, ‘Frequency and antibiogram of multi-drug resistant Pseudomonas aeruginosa’, *Journal of the College of Physicians and Surgeons: Pakistan* 21(9), 531–534. 21914408

[CIT0015] Hauser A.R. & Ozer E.A., 2011, ‘Pseudomonas aeruginosa’, *Nature Reviews Microbiology* 9(3), 2011. 10.1038/nrmicro2199 PMC2766515 19680249

[CIT0016] Iregbu K. & Eze S., 2015, ‘Pseudomonas aeruginosa infections in a tertiary hospital in Nigeria’, *African Journal of Clinical and Experimental Microbiology* 16(1), 33–36. 10.4314/ajcem.v16i1.6

[CIT0017] Javiya V., Ghatak S., Patel K. & Patel J., 2008, ‘Antibiotic susceptibility patterns of Pseudomonas aeruginosa at a tertiary care hospital in Gujarat, India’, *Indian Journal of Pharmacology* 40(5 suppl), 230–234. 10.4103/0253-7613.44156 20040963 PMC2792624

[CIT0018] Khan F., Khan A. & Kazmi S.U., 2014, ‘Prevalence and susceptibility pattern of multi drug resistant clinical isolates of Pseudomonas aeruginosa in Karachi’, *Pakistan Journal of Medical Sciences* 30(5), 951–954. 10.12669/pjms.305.5400 25225505 PMC4163210

[CIT0019] Khan M.A. & Faiz A., 2016, ‘Antimicrobial resistance patterns of Pseudomonas aeruginosa in tertiary care hospitals of Makkah and Jeddah’, *Annals of Saudi Medicine* 36(1), 23–28. 10.5144/0256-4947.2016.23 26922684 PMC6074268

[CIT0020] Magiorakos A., Srinivasan A., Carey R.B., Carmeli Y., Falagas M.E., Giske C.G. et al., 2011, ‘Bacteria : An international expert proposal for interim standard definitions for acquired resistance’, *Clinical Microbiology and Infection* 18(3), 268–281. 10.1111/j.1469-0691.2011.03570.x 21793988

[CIT0021] Marza J.A.S., Soothill J.S., Boydell P. & Collyns T.A., 2006, ‘Multiplication of therapeutically administered bacteriophages in Pseudomonas aeruginosa infected patients’, *Burns* 32(5), 644–646. 10.1016/j.burns.2006.02.012 16781080

[CIT0022] Mekić S., Matanović K. & Šeol B., 2011, ‘Antimicrobial susceptibility of Pseudomonas aeruginosa isolates from dogs with otitis externa’, *Veterinary Record* 169(5), 125 10.1136/vr.d2393 21742683

[CIT0023] Mishra S.K., Acharya J., Kattel H.P., Koirala J., Rijal B.P. & Pokhrel B.M., 2012, ‘Metallo-beta-lactamase producing gram-negative bacterial isolates’, *Journal of Nepal Health Research Council* 10(22), 208–213. 23281453

[CIT0024] Mokaddas E.M. & Sanyal S.C., 1999, ‘Resistance patterns of Pseudomonas aeruginosa to carbapenems and piperacillin/tazobactam’, *Journal of Chemotherapy* 11(2), 93–96. 10.1179/joc.1999.11.2.93 10326738

[CIT0025] Morita Y., Kimura N., Mima T., Mizushima T. & Tsuchiya T., 2001, ‘Roles of MexXY- and MexAB-multidrug efflux pumps in intrinsic multidrug resistance of Pseudomonas aeruginosa PAO1’, *The Journal of General and Applied Microbiology* 47(1), 27–32. 10.2323/jgam.47.27 12483565

[CIT0026] Morita Y., Tomida J. & Kawamura Y., 2013, ‘Responses of Pseudomonas aeruginosa to antimicrobials’, *Frontiers in Microbiology* 4(422), 422 10.3389/fmicb.2013.00422 PMC3884212 24409175

[CIT0027] Mudau M., Jacobson R., Minenza N., Kuonza L., Morris V., Engelbrecht H. et al., 2013, ‘Outbreak of multi-drug resistant Pseudomonas aeruginosa bloodstream infection in the haematology unit of a South African Academic Hospital’, *PLoS One* 8(3), 1–7. 10.1371/journal.pone.0055985 PMC3597724 23516393

[CIT0028] Musser A.W. & Beamer P.R., 1961, ‘Infections caused by Pseudomonas aeruginosa’, *The Journal of the Indiana State Medical Association* 54(2), 1627–1634. 10.1097/00007611-196102000-00036 14477572

[CIT0029] Nicas T.I. & Hancock R.E.W., 1983, ‘Pseudomonas aeruginosa outer membrane permeability: Isolation of a porin protein F-deficient mutant’, *Journal of Bacteriology* 153(1), 281–285. 10.1128/JB.153.1.281-285.1983 6294050 PMC217367

[CIT0030] Odjadjare E.E., Igbinosa E.O., Mordi R., Igere B., Igeleke C.L. & Okoh A.I., 2012, ‘Prevalence of multiple antibiotics resistant (MAR) Pseudomonas species in the final effluents of three municipal wastewater treatment facilities in South Africa’, *International Journal of Environmental Research and Public Health* 9(6), 2092–2107. 10.3390/ijerph9062092 22829792 PMC3397366

[CIT0031] Oliveira L.C., Medeiros C.M.O., Silva I.N.G., Monteiro A.J., Leite C.A.L. & Carvalho C.B.M., 2005, ‘Susceptibilidade a antimicrobianos de bactérias isoladas de otite externa em cães’, *Arquivo Brasileiro de Medicina Veterinária e Zootecnia* 57(3), 405–408. 10.1590/S0102-09352005000300021

[CIT0032] Pang Z., Raudonis R., Glick B.R., Lin T.J. & Cheng Z., 2019, ‘Antibiotic resistance in Pseudomonas aeruginosa: Mechanisms and alternative therapeutic strategies’, *Biotechnology Advances* 37(1), 177–192. 10.1016/j.biotechadv.2018.11.013 30500353

[CIT0033] Papp-Wallace K.M., Endimiani A., Taracila M.A. & Bonomo R.A., 2011, ‘Carbapenems: Past, present, and future’, *Antimicrobial Agents and Chemotherapy* 55(11), 4943–4960. 10.1128/AAC.00296-11 21859938 PMC3195018

[CIT0034] Pechère J.C. & Köhler T., 1999, ‘Patterns and modes of β-lactam resistance in Pseudomonas aeruginosa’, *Clinical Microbiology and Infection* 5(suppl 1), S15–S18. 10.1111/j.1469-0691.1999.tb00719.x 11869272

[CIT0035] Peleg A.Y. & Hooper D.C., 2010, ‘Hospital-acquired infections due to gram-negative bacteria’, *New England Journal of Medicine* 362(19), 1804–1813. 10.1056/NEJMra0904124 20463340 PMC3107499

[CIT0036] Penna B., Thomé S., Martins R., Martins G. & Lilenbaum W., 2011, ‘In vitro antimicrobial resistance of Pseudomonas aeruginosa isolated from canine otitis externa in Rio de Janeiro, Brazil’, *Brazilian Journal of Microbiology* 42(4), 1434–1436. 10.1590/S1517-83822011000400027 24031774 PMC3768740

[CIT0037] Perovic O., Koornhof H.J., Crewe-Brown H.H., Duse A.G., van Nierop W. & Galpin J.S., 2008, ‘Pseudomonas aeruginosa bacteraemia in an academic hospital in South Africa’, *South African Medical Journal* 98(8), 626–632. 18928043

[CIT0038] Petrov V., Mihaylov G., Tsachev I., Zhelev G., Marutsov P. & Koev K., 2013, ‘Otitis externa in dogs: Microbiology and antimicrobial susceptibility’, *Revue de Medecine Veterinaire* 164(1), 18–22.

[CIT0039] Pintarić S, Matanović K. & Martinec B.Š., 2017, ‘Fluoroquinolone susceptibility in Pseudomonas aeruginosa isolates from dogs: Comparing disk diffusion and microdilution methods’, *Veterinarski Arhiv* 87(3), 291–300. 10.24099/vet.arhiv.160120

[CIT0040] Poole K., 2005, ‘Aminoglycoside resistance in Pseudomonas aeruginosa’, *Antimicrobial Agents and Chemotherapy* 49(2), 479–487. 10.1128/AAC.49.2.479-487.2005 15673721 PMC547279

[CIT0041] Prescott J.F., Giguere S., Baggot J., Walker R. & Dowling P., 2003, ‘Antimicrobial therapy in veterinary medicine. 4th edition’, *Canadian Veterinary Journal Revue Veterinaire Canadienne* 44(February), 7817.

[CIT0042] Pye C., 2018, ‘Pseudomonas otitis externa in dogs’, *The Canadian Veterinary Journal = La Revue Veterinaire Canadienne* 59(11), 1231–1234. 30410185 PMC6190182

[CIT0043] Quinn P.J., Carter M.E., Markey B. & Carter G.R., 1994, *Clinical veterinary microbiology*, Mosby Wolfe, Edinburgh.

[CIT0044] Rafiee R., Eftekhar F., Tabatabaei S.A. & Tehrani D.M., 2014, ‘Prevalence of extended-spectrum and metallo β-lactamase production in AmpC β-lactamase producing Pseudomonas aeruginosa isolates from burns’, *Jundishapur Journal of Microbiology* 7(9), e16436 10.5812/jjm.16436 25485066 PMC4255381

[CIT0045] Rubin J., Walker R.D.D., Blickenstaff K., Bodeis-Jones S. & Zhao S., 2008, ‘Antimicrobial resistance and genetic characterization of fluoroquinolone resistance of Pseudomonas aeruginosa isolated from canine infections’, *Veterinary Microbiology* 131(1–2), 164–172. 10.1016/j.vetmic.2008.02.018 18395369

[CIT0046] Shah D.A., Wasim S. & Abdullah F.E., 2015, ‘Antibiotic resistance pattern of Pseudomonas aeruginosa isolated from urine samples of urinary tract infections patients in Karachi, Pakistan’, *Pakistan Journal of Medical Sciences* 31(2), 341–345. 26101487 10.12669/pjms.312.6839 PMC4476338

[CIT0047] Shokri D., Rabbani Khorasgani M., Zaghian S., Fatemi S.M., Mohkam M., Ghasemi Y. et al., 2016, ‘Determination of acquired resistance profiles of Pseudomonas aeruginosa isolates and characterization of an effective bacteriocin-like inhibitory substance (BLIS) against these isolates’, *Jundishapur Journal of Microbiology* 9(8), e32795 10.5812/jjm.32795 27800131 PMC5080677

[CIT0048] Steen S.I. & Paterson S., 2012, ‘The susceptibility of Pseudomonas spp. isolated from dogs with otitis to topical ear cleaners’, *Journal of Small Animal Practice* 53(10), 599–603. 10.1111/j.1748-5827.2012.01262.x 22889046

[CIT0049] Strateva T. & Yordanov D., 2009, ‘Pseudomonas aeruginosa: A phenomenon of bacterial resistance’, *Journal of Medical Microbiology* 58(9), 1133–1148. 10.1099/jmm.0.009142-0 19528173

[CIT0050] Tam V.H., Chang K.T., Abdelraouf K., Brioso C.G., Ameka M., McCaskey L.A. et al., 2010, ‘Prevalence, resistance mechanisms, and susceptibility of multidrug-resistant bloodstream isolates of Pseudomonas aeruginosa’, *Antimicrobial Agents and Chemotherapy* 54(3), 1160–1164. 10.1128/AAC.01446-09 20086165 PMC2826008

[CIT0051] Thompson M.F., Litster A.L., Platell J.L. & Trott D.J., 2011, ‘Canine bacterial urinary tract infections: New developments in old pathogens’, *Veterinary Journal* 190(1), 22–27. 10.1016/j.tvjl.2010.11.013 21239193

[CIT0052] Türkyilmaz S., 2008, ‘Antibiotic susceptibility patterns of Pseudomonas aeruginosa strains isolated from dogs with otitis externa’, *Turkish Journal of Veterinary and Animal Sciences* 32(1), 37–42.

[CIT0053] Ullah F., Malik S.A. & Ahmed J., 2009, ‘Antimicrobial susceptibility and ESBL prevalence in Pseudomonas aeruginosa isolated from burn patients in the North West of Pakistan’, *Burns* 35(7), 1020–1025. 10.1016/j.burns.2009.01.005 19501980

[CIT0054] Vingopoulou E.I., Delis G.A., Batzias G.C., Kaltsogianni F., Koutinas A., Kristo I. et al., 2018, ‘Prevalence and mechanisms of resistance to fluoroquinolones in Pseudomonas aeruginosa and Escherichia coli isolates recovered from dogs suffering from otitis in Greece’, *Veterinary Microbiology* 213(October 2017), 102–107. 10.1016/j.vetmic.2017.11.024 29291992

[CIT0055] Weese J.S., Blondeau J., Boothe D., Guardabassi L.G., Gumley N., Papich M. et al., 2019, ‘International society for companion animal infectious diseases (ISCAID) guidelines for the diagnosis and management of bacterial urinary tract infections in dogs and cats’, *Veterinary Journal* 247, 8–25. 10.1016/j.tvjl.2019.02.008 30971357

[CIT0056] Wong C., Epstein S.E. & Westropp J.L., 2015, ‘Antimicrobial susceptibility patterns in urinary tract infections in dogs (2010-2013)’, *Journal of Veterinary Internal Medicine* 29(4), 1045–1052. 10.1111/jvim.13571 26133165 PMC4895361

[CIT0057] Yukawa S., Tsuyuki Y., Sato T., Fukuda A., Usui M. & Tamura Y., 2017, ‘Antimicrobial resistance of Pseudomonas aeruginosa isolated from dogs and cats in primary veterinary hospitals in Japan’, *Japanese Journal of Infectious Diseases* 70(4), 461–463. 10.7883/yoken.JJID.2016.536 28367887

